# Antidiabetic and Renoprotective Effects of *Coffea arabica* Pulp Aqueous Extract through Preserving Organic Cation Transport System Mediated Oxidative Stress Pathway in Experimental Type 2 Diabetic Rats

**DOI:** 10.3390/molecules26071907

**Published:** 2021-03-28

**Authors:** Oranit Boonphang, Atcharaporn Ontawong, Tipthida Pasachan, Manussabhorn Phatsara, Acharaporn Duangjai, Doungporn Amornlerdpison, Metee Jinakote, Chutima Srimaroeng

**Affiliations:** 1 Department of Physiology, Faculty of Medicine, Chiang Mai University, Chiang Mai 50200, Thailand; oranit795@gmail.com (O.B.); tp.pasachan@gmail.com (T.P.); 2 Division of Physiology, School of Medical Sciences, University of Phayao, Phayao 56000, Thailand; atcharaporn.on@up.ac.th (A.O.); achara.phso@gmail.com (A.D.); 3 Department of Anatomy, Faculty of Medicine, Chiang Mai University, Chiang Mai 50200, Thailand; msethadavit@gmail.com; 4 Centre of Excellence in Agricultural Innovation for Graduate Entrepreneur and Faculty of Fisheries Technology and Aquatic Resources, Maejo University, Chiang Mai 50290, Thailand; doungpornfishtech@gmail.com; 5 School of Human Kinetics and Health, Faculty of Health Science Technology, HRH Princess Chulabhorn College of Medical Science, Chulabhorn Royal Academy, Bangkok 10210, Thailand; metee.jin@cra.ac.th

**Keywords:** antidiabetic effect, antioxidant, *Coffea arabica* pulp, diabetes mellitus, renal organic cation transport

## Abstract

*Coffea arabica* pulp (CP) is a by-product of coffee processing. CP contains polyphenols that have exhibited beneficial effects, including antioxidant and lipid-lowering effects, as well as enhanced insulin sensitivity, in in vitro and in vivo models. How polyphenols, as found in CP aqueous extract (CPE), affect type 2 diabetes (T2D) has not been investigated. Thus, the present study examined the potential antidiabetic, antioxidant, and renoprotective effects of CPE-rich polyphenols, using an experimental model of T2D in rats induced by a high-fat diet and a single low dose of streptozotocin. The T2D rats received either 1000 mg/kg body weight (BW) of CPE, 30 mg/kg BW of metformin (Met), or a combination treatment (CPE + Met) for 3 months. Plasma parameters, kidney morphology and function, and renal organic transport were determined. Significant hyperglycemia, hypertriglyceridemia, insulin resistance, increased renal lipid content and lipid peroxidation, and morphological kidney changes related to T2D were restored by both CPE and CPE + Met treatments. Additionally, the renal uptake of organic cation, ^3^H-1-methyl-4-phenylpyridinium (MPP^+^), was reduced in T2D, while transport was restored by CPE and CPE + Met, through an up-regulation of antioxidant genes and protein kinase Cα deactivation. Thus, CPE has antidiabetic and antioxidant effects that potentially ameliorate kidney function in T2D by preserving renal organic cation transport through an oxidative stress pathway.

## 1. Introduction

Type 2 diabetes mellitus (T2D) patients are the largest group of diabetic patients; this disease is characterized by hyperglycemia, insulin resistance, and impaired insulin secretion [1]. Long-term hyperglycemia leads to tissue and organ damage as well as the subsequent development of diabetic complications, including retinopathy, neuropathy, and diabetic nephropathy (DN) [1,2]. A previous study reported that 44% of T2D patients have progressively diminishing renal function, which leads to further DN and end-stage renal disease [3]. Additionally, DN is caused by the excessive generation of reactive oxygen species, which ultimately induces oxidative stress in renal tissue [4]. Therefore, an antioxidant exhibiting renoprotective effects could help improve or delay the progression of DN that is induced by oxidative stress.

DN decreases renal function through altering glomerular filtration, resulting in microalbuminuria and impaired renal tubular secretory processes [3,5,6]. It is known that the renal tubular secretion of organic anions and cations, uremic toxins, xenobiotics, and heavy metals, is primarily mediated by specific transporters concentrated on the basolateral membrane of the renal proximal tubules, including organic anion transporter 1 (Oat1), organic anion transporter 3 (Oat3), and organic cation transporter 2 (Oct2) [7,8]. The regulation of organic drug secretion and, thus, drug pharmacokinetics is dependent of these transporters, particularly in patients prescribed multiple drugs [8,9]. The impairment of these transporters, therefore, can lead to the altered renal clearance of substrates and, subsequently, organic ion accumulation and nephrotoxicity. Previous studies have demonstrated that the down-regulation of organic cation transporter 1 (Oct1) and Oct2 expression and function in streptozotocin induced-diabetic rats is associated with transcriptional and/or translational alteration, increased advanced glycation end-products, and activation of the renin-angiotensin system [10,11]. Meanwhile, grape seed, rich in procyanidins, has demonstrated antioxidant effects and increased intestinal transport of organic cation 1-methyl-4-phenylpyridinium (MPP^+^) in human colon carcinoma cells [12]. Recently, oxidative stress has been shown to induce increased protein kinase Cα (PKCα) activation and impaired insulin-stimulated Oat3 transport function in the T2D rat kidney; these effects were reversed by an antioxidant polyphenol-rich *Spirogyra neglecta* [13]. Nevertheless, there is limited information regarding the renoprotective effects of antioxidants on renal organic cation transport in T2D.

The infusion of *Coffea arabica* L. (Rubiaceae) is the most popular beverage worldwide; meanwhile, this industry produces large amounts of solid residues from coffee berries, called *Coffea arabica* pulp (CP) [14]. Fresh CP contains antioxidative phenolic compounds, including chlorogenic acid (CGA), epicatechin, catechin, caffeine, and anthocyanins [15]. A previous study showed that CG A not only decreases fasting plasma cholesterol, triglycerides, and hepatic lipid accumulation but also improves glucose tolerance and insulin sensitivity in obese (db/db) mice [16]. Moreover, anthocyanin demonstrates antioxidant, anti-inflammatory, anticancer, and antiaging properties [14,17]. More recently, our studies reported that CGA, caffeine, epicatechin, and catechin-rich in CP aqueous extract (CPE) exhibited antiobesity and hepatoprotective effects in high-fat diet-induced obese rats [18,19]. Whether CPE has antidiabetic and antioxidant effects on renal transport function in T2D has not yet been investigated. Therefore, the possibility of a protective effect of CPE on renal organic cation and anion transporter function, as well as potential mechanisms, are addressed in this study on experimental T2D rats.

## 2. Results

### 2.1. Effect of CPE on Selected Overall Nutritional, Growth, and Biochemical Parameters of Carbohydrate and Lipid Metabolism as Well as General Kidney Function in Rats

As shown in Table 1, diabetic rats had significantly higher daily food and calorie intake compared to ND rats. However, food intake in DM + CPE and DM + CPE + Met rats did not differ when compared with ND rats, while the calorie intake in these groups was at similar levels compared to that of DM rats. Water intake, BW, and relative kidney weight were not significantly different among experimental groups. Interestingly, ND + CPE rats had significantly less BW gain and visceral fat weight per BW (VFW/BW), whereas, DM rats had markedly higher VFW/BW compared to ND rats. Additionally, DM + CPE and DM + CPE + MET rats had lower VFW/BW compared to DM rats, suggesting that CPE prevents fat deposition and controls BW under normal and T2D conditions.

Similar to T2D characteristics in humans, DM rats had marked hyperglycemia, hypertriglyceridemia, and insulin resistance, reflected by a higher HOMA index when compared to ND rats. DM + CPE, DM + Met, and DM + CPE + Met rats demonstrated significantly ameliorated glucose levels, triglyceride levels, and insulin resistance when compared to DM rats; meanwhile, insulin levels and the eGFR, which represents renal function, were not significantly different among the experimental groups (Table 2). As such, CPE demonstrates antidiabetic effects via decreasing plasma glucose, triglycerides, and insulin resistance in early T2D without affecting general renal function.

### 2.2. Effect of CPE on Pathological Renal Damage

**Figure 1:**
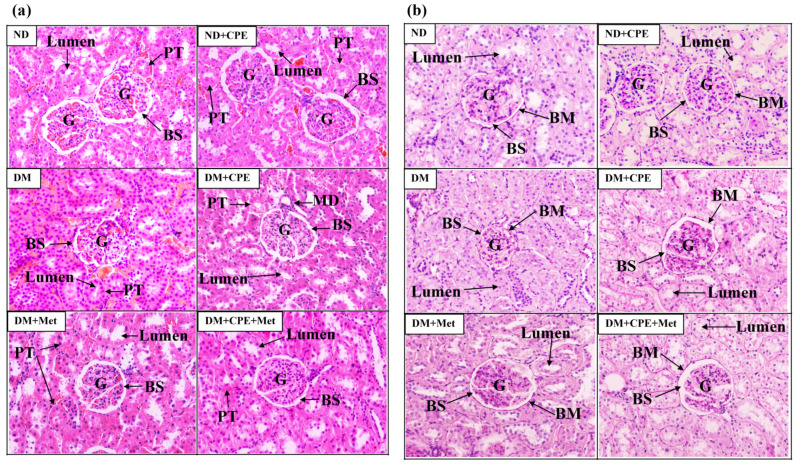
Effect of CPE on renal histopathological photomicrograph in T2D rats stained by (**a**) hematoxylin and eosin and (**b**) periodic acid-Schiff base at 200×X. The data were taken from 5–6 different animals and analyzed using bright-field microscopy. Arrow “BS” indicates the Bowman’s capsule space; “LS” indicates the tubular lumen space; “PT” indicates the proximal tubular epithelial cells and proximal convoluted tubule; “MD” indicates the macular densa; and “BM” indicates the basement membrane. Abbreviation: glomerulus (G).

As shown in Figure 1a, there were no differences in the glomerular and tubular structures of ND and ND + CPE rats, whereas DM rat kidneys showed pathological lesions, specifically glomerular hypertrophy, narrowing of the Bowman’s capsule space, and narrowing of the tubular lumen, when compared to ND rat kidneys. In contrast, DM + CPE rats displayed a reversal of the renal pathological damage, shown by wider Bowman’s capsule spaces and wider proximal tubular lumens, similar to those of DM + Met and DM + CPE + Met rats. Consistently, PAS staining showed a mild degree of mesangial expansion, glomerular and tubular basement membrane thickening, and renal tubular lumen narrowing in DM rat kidneys when compared to ND and ND + CPE rat kidneys (Figure 1b). In the renal tissue of DM + CPE, DM + Met, and DM + CPE + Met rats, these renal pathologies were not present (Figure 1b), indicating that CPE had no nephrotoxic effect.

### 2.3. Effect of CPE on Renal Lipid Content and Lipid Peroxidation

**Figure 2:**
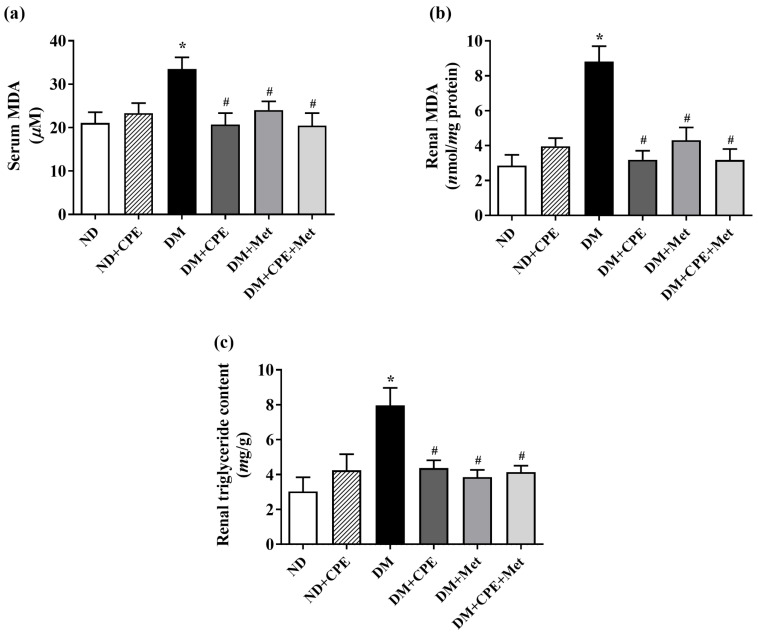
Effect of CPE on oxidative stress determined by lipid peroxidation as indicated by (**a**) serum malondialdehyde (MDA), (**b**) renal cortical MDA, and (**c**) total renal triglyceride content in T2D rats. Data are expressed as mean ± S.E.M (*n* = 6). * *p* < 0.05, significantly different from ND; ^#^ *p* < 0.05, significantly different from DM.

Since triglyceride accumulation in the renal proximal tubules contributes to renal lipid peroxidation and oxidative stress in a model of T2D [20,21], we determined the total triglyceride content in the renal cortical tissues. As shown in Figure 2a,b, malondialdehyde levels in plasma and renal cortical tissues were markedly higher in the DM group, while the DM + CPE, DM + Met, and DM + CPE + Met rats displayed lower levels. Congruently, the total renal triglyceride was significantly higher in the DM rats when compared to ND rats. On the other hand, lower total triglyceride contents were observed in DM + CPE, DM + Met, and DM + CPE + Met rat kidneys (Figure 2c). These results indicate that CPE possesses antioxidant and renoprotective effects against systemic and renal lipid peroxidation, as well as protective effects against high levels of renal lipid accumulation.

### 2.4. Effect of CPE on Impairment of Renal Organic Cation Transport Function

**Figure 3:**
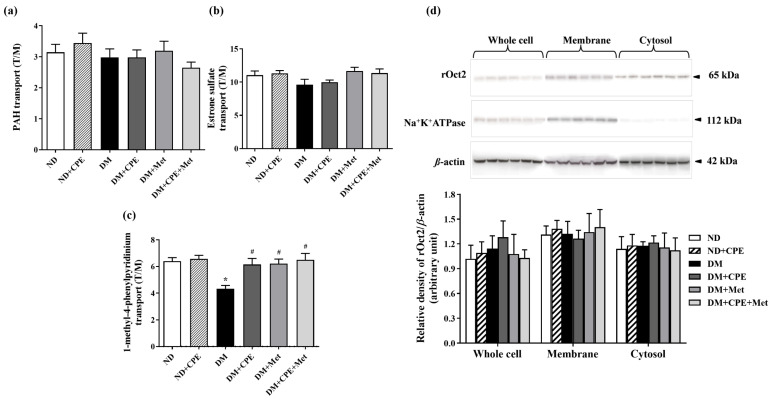
Effect of CPE on (**a**) *para*-aminohippurate transport mediated by rOat1 and 3, (**b**) estrone sulfate transport mediated by rOat3, and (**c**) 1-methyl-4-phenylpyridinium transport mediated by rOct2 in T2D rats. Data are expressed as tissue to medium ratios (T/M), i.e., tissue content (dpm/mg) ÷ medium (dpm/µL). Each experiment was performed in separate animals using 3 slices from each animal (*n* = 5–6). * *p* < 0.05, significantly different from ND; ^#^ *p* < 0.05, significantly different from DM. (**d**) Effect of CPE on rOct2 protein expression from rat kidney cell lysate, membrane, and cytosolic fractions, respectively. Anti-rOct2 antibody was detected at 65 kDa. Anti-Na^+^-K^+^-ATPase antibody at 112 kDa and anti-β-actin antibody at the 42 kDa were used as a membrane marker and loading control, respectively. Data are expressed as mean ± S.E.M. and repeated in separate animals (*n* = 4–5). A representative blot of rOct2 is shown in the top panel, and quantification of relative protein expression in each fraction is shown in the bottom panel.

Using ^3^H-PAH (substrate for Oat1 and Oat3), ^3^H-ES (specific substrate for Oat3), and ^3^H-MPP^+^ (substrate for Oct2), we further explored CPE’s effects on renal organic ion transport, as mediated by basolateral transporters. As shown in Figure 3a,b, the uptake of PAH and ES substrates was not significantly different among the experimental groups. Interestingly, renal slices from DM rats had significantly decreased MPP^+^ transport mediated by rOct2 when compared to ND rats (Figure 3c), while DM + CPE, DM + Met, and DM + CPE + Met rats displayed marked improvements in MPP^+^ transport. These findings suggest that the renoprotective effect of CPE on organic cation transport mediated by rOct2 in early T2D is similar to that of treatment with metformin alone or in combination with CPE. To further identify whether membrane levels of rOct2 protein are involved in CPE’s renoprotective effect, cellular rOct2 protein expression was investigated. Results showed that the rOct2 protein expression in each fraction was not different among experimental groups when compared with the respective control (ND) (Figure 3d), indicating that the reduced MPP^+^ transport mediated by rOct2 in T2D rats was not dependent on functional Oct2 protein expression.

### 2.5. Effect of CPE on Renal Antioxidative Gene Expression

**Figure 4:**
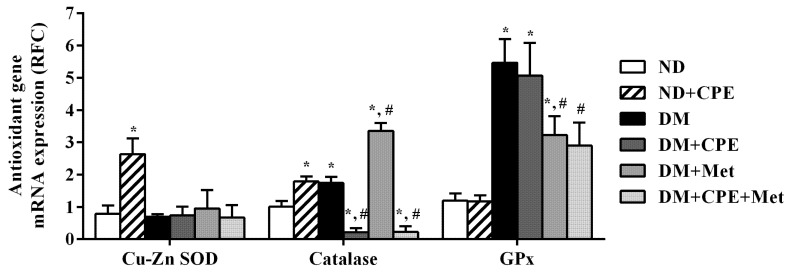
Effect of CPE on mRNA expression of renal antioxidant gene markers in T2D rats. Levels of Cu-Zn superoxide dismutase (Cu-Zn SOD), catalase, and glutathione peroxidase (GPx) mRNA were determined from total RNA extracted from rat renal cortical tissues using real-time quantitative PCR. The results are expressed as mean ± S.E.M and repeated in separate animals (*n* = 4–5). * *p* < 0.05, significantly different from ND; ^#^ *p* < 0.05, significantly different from DM.

As shown in Figure 4, the relative amplicon product of copper-zinc superoxide dismutase (Cu-Zn SOD) and catalase was significantly higher in ND + CPE rats when compared to ND rats, demonstrating CPE’s antioxidant properties through inducing Cu-Zn SOD and catalase gene transcription. Additionally, catalase transcripts were markedly higher in DM rat kidneys and, to an even greater extent, DM + Met rat kidneys, when compared to ND rats. In contrast, DM + CPE and DM + CPE + Met rats had significantly lower catalase transcripts when compared to ND and DM rats. Furthermore, glutathione peroxidase (GPx) mRNA expression in DM, DM + CPE, and DM + Met rats was significantly higher than in ND rats, while DM + Met and DM + CPE + Met rats reduced GPx expression when compared to DM rats. The findings indicate that the experimental model of T2D in rats had no effect on the expression of Cu-Zn SOD mRNA, but did up-regulate the expression of catalase and GPx genes. Similar to its effect on renal lipid peroxidation, CPE has renoprotective effects against oxidative stress via directly activating catalase and GPx gene expression in renal epithelial cells, thereby improving renal transport function in T2D.

### 2.6. Effect of CPE on Stress-Sensitive Signaling

**Figure 5:**
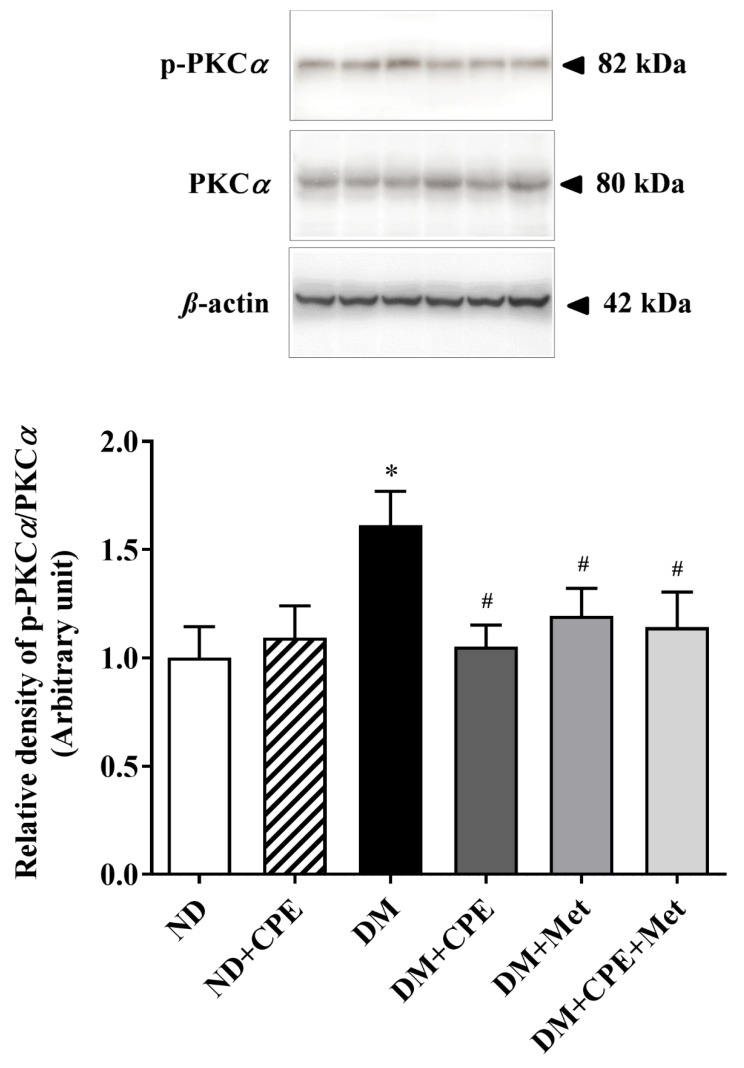
Effect of CPE on PKCα expression and activation, which is determined by p-PKCα/PKCα ratio in T2D rat kidney cell lysate. Anti-p-PKCα and PKCα antibodies were detected at 82 and 80 kDa, respectively, whereas anti-β-actin antibody was used as a loading control. Densitometry was analyzed and expressed as mean ± S.E.M., and the experiment was repeated in animals (*n* = 4–5). * *p* < 0.05, significantly different from ND; ^#^ *p* < 0.05, significantly different from DM.

To identify mechanisms involved in improved MPP^+^ transport by CPE, the expression of the stress-sensitive signaling protein, PKCα, was determined. As shown in Figure 5, the expression of p-pKCα relative to PKCα was markedly higher in DM rat kidneys when compared to ND rats. In contrast, the expression of p-PKCα/PKCα was significantly lower in DM + CPE, DM + Met, and DM + CPE + Met rats, suggesting that CPE had renoprotective effects partly via inhibiting the stress-sensitive signaling pathway that, in turn, improved organic cation transport mediated by rOct2.

## 3. Discussion

T2D is characterized by excessive hepatic glucose production, a decrease in insulin secretion, and insulin resistance [1]. Furthermore, insulin resistance is associated with several metabolic abnormalities, such as central obesity, hypertension, and dyslipidemia, which contribute to a high rate of cardiovascular morbidity and mortality [22]. In this study, CPE alone and in combination with metformin exhibited antidiabetic effects by decreasing plasma glucose and triglycerides, and improving insulin resistance. These effects were similar to those found from the action of metformin, which is a first-line drug for patients with T2D. The major constituents in CPE, specifically CGA and catechin, have been shown to have antidiabetic effects in db/db and T1D rats [23,24]. Interestingly, this study showed an antifat deposition effect of CPE, similar to the observed effects of CGA and catechin, which have been demonstrated to result in reduced abdominal adipose tissues in rodents and humans, respectively [23,25]. Recent have studies also demonstrated improved lipid metabolism resulting from CGA administration via AMP-dependent kinase, in leptin receptor knock-out mice [16,23]. Accordingly, increases in visceral adipose tissue are closely associated with the progression of T2D via the activation of systemic inflammation, resulting in insulin resistance [3,26]. In the present study, CPE also improved peripheral insulin resistance, indicating that CPE could restore insulin resistance by preventing abdominal fat deposition.

DN affects 44% of diabetic patients and is characterized by the progressive deterioration of renal function [3,27]. Moreover, approximately 50% of diabetic patients have end-stage renal disease associated, mainly, with proximal tubular structural alterations [3,27,28]. Consistently, this study revealed glomerular hypertrophy, mesangial expansion, and basement membrane thickening in both the glomerular and tubular portions of the nephron in high-fat diet-induced T2D rats, similar to previous findings [27]. Again, CPE alone or in combination with metformin reduced renal injury similar to that of metformin alone, suggesting that CPE has renoprotective effects against DN. Similarly, CGA, a major constituent of CPE, remarkably alleviated renal histopathological damage in 5/6-nephrectomized rats and mice with D-galactose-induced kidney injury [29,30].

In addition to long-term hyperglycemic exposure, renal lipid deposition is a cause of lipotoxicity, which is an additional mechanism of DN pathogenesis [31]. Recently, renal triglyceride content has been associated with the dysregulation of lipid metabolism in DN [3]. Hyperglycemia induces sterol regulatory element-binding protein-1 (SREBP-1) expression; this transcription factor activates fatty acid synthase, which increases the synthesis and accumulation of triglycerides in the kidney, as demonstrated in several models, including STZ-induced diabetic rats, SREBP-1 transgenic mice, murine cortical tubular cells, and other T2D models [21,32]. Additionally, hyperglycemia impairs renal cholesterol homeostasis by down-regulating cholesterol efflux transporters, adenosine triphosphate binding cassette (ABC) transporter A1 (ABCA1), ABC transporter G1 (ABCG1), and scavenger receptor class B type I, while increasing the cluster of differentiation 36, CD36, which, in turn, activates lipogenesis [33]. Consistently, this study showed that total renal triglyceride content was markedly higher in T2D rats, while rats supplemented with polyphenols, rich in CPE displayed lower total renal triglyceride levels. Thus, CPE may modulate the function of transport proteins, leading to reduced renal triglyceride accumulation, lipid peroxidation, and oxidative stress in renal epithelial cells. Like CPE, curcumin decreases renal lipid accumulation in T2D rats, while rockweed containing CGA (*Pilea microphylla* (L.)) decreases renal lipid peroxidation in high-fat diet- and STZ-induced T2D mice [21,34].

Persistent hyperglycemia and hyperlipidemia lead to the overproduction of free radicals, which induces oxidative stress that is known to initiate DN development [3,35]. Modifying antioxidant genes is known to potently defend against oxidative stress [36]. Consistently, CPE has demonstrated robust renoprotective effects against oxidative stress by modifying the mRNA expression of major antioxidant genes, specifically Cu-Zn SOD and catalase. CGA and epicatechin, polyphenols found in CPE, have been shown to protect osteoblast cells and brain tissue from hydrogen peroxide-induced oxidative stress and intracerebral hemorrhage, respectively, through the antioxidant and nuclear factor-erythroid 2-related factor 2 pathways [35,36]. CGA promotes the expression and activity of SOD, catalase, and GPx in an isoproterenol-induced oxidative stress model in myocardium and 5/6-nephrectomized rats [30,37]. Moreover, metformin attenuates the oxidative stress induced by carbon tetrachloride in mouse livers through enhanced catalase activity [38].

Several substances, including endogenous organic anions and cations, drugs, xenobiotics, heavy metals, and uremic toxins, are secreted into the proximal tubular lumen from the blood circulation via specific transporters, including Oat1, Oat3, and Oct2. These transporters are highly expressed on basolateral renal proximal tubules [39]. Thus, the impairment of these transporters via decreasing their expression or reducing their activity could potentially alter the renal clearance of organic substances, causing accumulation and nephrotoxicity. Although no changes to the organic anion transport system were observed in this study, a decrease in renal organic cation transport function was observed in T2D without alterations in membrane transporter expression. Similarly, the function of rOct1 and rOct2 has been shown to be decreased in STZ-induced T1D rats [11]. As the regulation of renal organic cation transport function is complex, involving isoform- and response-specific modulation [40], rOct2 function may concomitantly contribute to decreased MPP^+^ transport. Although rOct2 is highly expressed in the S2 and S3 segments of the renal proximal tubule of humans and rodents, rOct1 is also predominantly expressed in the S1 and S2 segments of the renal proximal convoluted tubules of rodents only [41]. Impaired rOct1 function, therefore, may also contribute to the decreased organic cation transport in this study. Additionally, p-PKC down-regulated human Oct2 in isolated human proximal tubules, while a mutation of cysteine at the extracellular loop of human Oct2 impaired human Oct2 protein folding, trafficking, and function [42,43]. Hence, the reduced organic cation transport in T2D in this study might be due to the oxidative stress-induced activation of PKCα, resulting in the further phosphorylation of intra- and/or extra-cellular loops of rOct2, thus interfering with its substrate binding site and decreasing MPP^+^ transport function. In support of this hypothesis, CPE, metformin, and the combination of CPE with metformin dephosphorylated PKCα, which, in turn, restored organic cation transport. Thus, the renoprotective effects of CPE on organic cation transport may be predominantly mediated via the oxidative stress/PKC activation pathway. Previous studies have shown that p-PKCα stimulates liver X receptor (LXR), while LXRα down-regulates rabbit Oct2 function [44,45]. Therefore, PKCα may subsequently activate LXRα, contributing to the reduced rOct2 function observed in this study.

## 4. Materials and Methods

### 4.1. Chemicals

Streptozotocin and CelLytic™ mammalian tissue lysis/extraction reagent were purchased from Sigma Aldrich (St. Louis, MO, USA). Polyclonal rabbit anti-Oct2 antibody was purchased from AlphaDiagnostics (San Antonio, TX, USA), and polyclonal rabbit anti-PKCα and phosphorylated PKCα (p-PKCα) were purchased from Santa Cruz Biotechnology (Santa Cruz, CA, USA). Monoclonal mouse anti-sodium potassium ATPase (Na^+^-K^+^-ATPase) and anti-β actin antibodies were obtained from Novus Biologicals (Littleton, CO, USA). Goat anti-mouse and rabbit immunoglobin G (IgG) horseradish peroxidase-conjugated secondary antibodies were obtained from EMD Millipore (Temecula, CA, USA). ^3^H-*para*-aminohippurate (PAH, specific activity (SA) 1 Ci/mmol) and ^3^H-estrone sulfate (ES, SA 50 Ci/mmol) were obtained from PerkinElmer Life Sciences (Boston, MA, USA). ^3^H-1-methyl-4-phenylpyridinium (MPP^+^, SA 80 Ci/mmol) was purchased from American Radiolabeled Chemicals, Inc. (St. Louis, MO, USA). All other chemicals with high purity were obtained from commercial sources.

### 4.2. Coffea arabica Pulp Aqueous Extract Preparation and Total Phenolic Content Measurement

CP was kindly provided by Hillkoff Co., Ltd. (Chiang Mai, Thailand). The voucher specimen is no. NU003806. CPE was extracted according to our recent report [19]. Additionally, phenolic contents, including chlorogenic acid, caffeine, catechin, and epicatechin, were also recently identified from our report [19]. Briefly, dried CP was ground, blended, and infused with boiled water at 100 °C for 10 min. CP aqueous solution was filtered through filter paper three times before being freeze-dried using a CoolSafe 110-4 Pro freeze dryer (Scanvac, Lillerød, Denmark), producing CPE with a yield of 7 % (*w*/*w*). Total phenolic content was quantified using Folin-Ciocalteu reagent, as previously described [14]. For quality control purposes, when polyphenol content reached a minimum of 22.72 ± 2.76 mg gallic acid equivalent/g extract, CPE was selected for further experiments.

### 4.3. Animals and Induction of Experimental Type 2 Diabetic Rats

Male Wistar rats, each weighing between 170 and 220 g, were obtained from the National Animal Center, Mahidol University, Salaya, Thailand. The animal facility and protocols were approved by the Laboratory Animal Care and Use Committee at the Faculty of Medicine, Chiang Mai University, Chiang Mai, Thailand (Protocol no.10/2559). All experimental rats were housed under a 12 h light/dark cycle. The rats were randomly divided into two groups: normal diet rats consumed a commercial normal chow diet (C.P. Mice Feed, no.082, Bangkok, Thailand) containing 19.77% total energy from fat (% E), while diet rats consumed food containing approximately 60% E total fat, ad libitum. Two weeks later, high-fat diet rats were intraperitoneally (i.p) injected with a single low dose of 35 mg/kg body weight (BW) of streptozotocin (STZ) (Sigma Aldrich, St. Louis, MO, USA) dissolved in 0.1 M citrate buffer; and normal diet rats received i.p. citrate buffer injection as previously described [46]. Two weeks after the injection, blood was collected via tail vein draws, and overnight fasting blood glucose levels were determined by colorimetric assay (Erba Diagnostics, Mannheim, Germany). Rats with a fasting blood glucose level exceeding 250 mg/dL were considered diabetic. Subsequently, the animals were randomly divided into 6 groups: normal diet (ND), ND supplemented with CPE at 1000 mg/kg BW (ND + CPE), T2D (DM), DM supplemented with CPE at 1000 mg/kg BW (DM + CPE), DM treated with metformin at 30 mg/kg BW (DM + Met), and DM receiving a combination of CPE at 1000 mg/kg BW and metformin at 30 mg/kg BW (DM + CPE + Met). The selection of this single, high dose was due to our recent study demonstrating that 1000 mg/kg BW of CPE contains approximately 12 mg/kg BW CGA [19]. Additionally, CGA, at a dose of 10 mg/kg BW, improves renal function and oxidative stress in DN rats [47], while metformin, at a dose of 30 mg/kg BW, exhibits antidiabetic effects in STZ-induced diabetic rats [48]. CPE and metformin were dissolved in deionized water. Rats received their respective supplement once daily for 12 weeks. Body weight was recorded weekly, while food and water intake were recorded daily. At the end of the experiment, 24 h urine volume was collected, and the animals were sacrificed.

### 4.4. Biochemical Analysis

Total plasma glucose and triglycerides were quantified by enzymatic colorimetric assay using commercial kits obtained from Erba Diagnostics (Mannheim, Germany). Plasma insulin concentrations were determined using Elisa assay kits from LINCO Research (Millipore, MA, USA). Homeostasis assessment of insulin resistance (HOMA index) was calculated to estimate insulin resistance using the following formula: fasting plasma insulin (µU/mL) × fasting plasma glucose (mmol/L) ÷ 22.5 [49]. Twenty-four-hour urine volume was recorded to calculate urine flow rate. Plasma and urine creatinine levels were measured using enzymatic kits obtained from Dialab (Wiener Neudorf, Austria), and the estimated glomerular filtration rate (eGFR) was subsequently calculated [50]. Serum and renal malondialdehyde (MDA) levels were measured using thiobarbituric acid assay kits (Cayman Chemical Company, Ann Arbor, MI, USA).

### 4.5. Histological Analysis

A quarter of each kidney was excised, fixed in 4% paraformaldehyde at 4 °C for 12–24 h, washed with PBS, and embedded in paraffin blocks. Subsequently, the paraffin blocks were cut into 5–7 μm thick sections and mounted on gelatin-coated slides. The slides were stained with either hematoxylin and eosin (H&E), for renal morphological assessment, or periodic acid-Schiff base (PAS), for elucidation of lesions as previously described [27]. The degree of severity was graded as mild, moderate, or severe when assessing focal changes with less than 25%, 25–50%, or greater than 50% lesions, respectively.

### 4.6. Renal Slice Preparation and Transport Study

To determine organic anion and cation transport function, the renal cortical tissues from the rats were sliced to 0.5 mm thickness using a Stadie-Riggs microtome as previously described [13]. The slices were incubated in freshly oxygenated ice-cold modified Cross and Taggart saline buffer containing 20 μM glutarate with 10 μM ^3^H-PAH (substrate for Oat1 and Oat3), 50 ηM ^3^H-ES (specific substrate for Oat3), or 1.25 ηM of ^3^H-MPP^+^ (substrate for Oct2) at room temperature for 30 min. At the end of the experiment, the reaction was stopped by the addition of 0.1 M MgCl_2_. The slices were blotted on Whatman no.1 paper, weighed, dissolved in 0.5 mL of 1 N NaOH, and neutralized with 0.5 mL of 1 N HCl. Radioactivity was quantified by liquid scintillation spectroscopy (Perkin Elmer, Waltham, MA, USA). Transport activity was calculated as the tissue to medium ratio (dpm/mg tissue ÷ dpm/µL medium).

### 4.7. Renal Triglyceride Accumulation and Lipid Peroxidation

Total renal cortical lipid content was measured using a modified method, as previously described [51]. Briefly, isolated rat cortical tissues were incubated, and total lipid contents were extracted by chloroform:isopropanol (7:11, *v*/*v*). Triglyceride levels were subsequently measured by enzymatic colorimetric assay as described above. For renal cortical MDA measurement, renal cortical tissue was cut and homogenized with CelLytic^TM^ MT lysis/extraction reagent (Sigma-Aldrich, St. Louis, MO, USA) containing 1% complete protease inhibitor cocktail (Millipore, Danvers, MA, USA), according to the manufacturer’s protocol, and centrifuged at 1600× *g* for 10 min at 4 °C. Renal tissue lysates were used to determine the total MDA level as described above. Data were normalized relative to mg protein.

### 4.8. Antioxidant Gene Expression Using Quantitative Real-Time Polymerase Chain Reaction (qPCR)

Total ribonucleic acid (RNA) was extracted and purified from freshly isolated rat renal cortical tissues using a total RNA extraction kit (Amresco, Columbus, OH, USA). Briefly, rat renal cortical tissue was homogenized in extraction reagent as per the manufacturer’s protocol. The purity and integrity of total RNA were determined by a multidetection microplate reader (Biotek, Winooski, VT, USA). Subsequently, first-strand complementary deoxyribonucleic acid (cDNA) was synthesized using the iScript™ cDNA synthesis kit (Bio-Rad, Hercules, CA, USA). mRNA levels were determined quantitatively using the Sensi-FAST SYBR Lo-Rox Kit (Bioline, London, UK). Primer sets were used as previously published [13] and were purchased from Integrated DNA Technologies (Coralville, IA, USA). Gene expressions were normalized to the β-actin mRNA level and expressed as relative fold changes (RFC). qPCR amplification was performed in duplicate for each cDNA.

### 4.9. Subcellular Fractions and Western Blot Analysis

Rat renal cortex samples were homogenized in lysis buffer containing 1 % complete protease inhibitor cocktail and centrifuged at 5000× *g* for 10 min at 4 °C. The supernatant from the homogenized samples was divided into 2 parts. The first part was designated as whole cell lysate, while the remaining portion was re-centrifuged at 100,000× *g* for 2 h. The supernatant fraction from this step was designated as the cytoplasmic fraction, while the pellet was re-suspended in the buffer and used as the membrane fraction. Total protein concentration from all samples was determined by Bradford protein assay (Bio-Rad, Hercules, CA, USA).

Protein samples were separated by resolving in 2×X Laemmli solution, subjecting them to electrophoresis on 10 % sodium dodecyl sulfate polyacrylamide gel, and transferring them onto polyvinylidene difluoride membranes (GE Healthcare, Waukesha, WI, USA). Non-specific binding was eliminated by blocking with 5% (*w*/*v*) non-fat dry milk in 0.05% Tween-20 in tris-buffered saline (T-TBS) for 1 h. The samples were then incubated overnight with specific primary antibody (rat Oct2 (rOct2), p-PKCα, PKCα). Membranes were washed with T-TBS buffer and incubated with a 1:10,000 dilution of horseradish peroxidase-conjugated ImmunoPure secondary goat anti-rabbit IgG or anti-mouse IgG (Millipore, Danvers, MA, USA) for 1 h. Proteins were detected using Super Signal West Pico Chemiluminescent Substrate (GE Healthcare, Waukesha, WI, USA) and quantitated by the Image J program version 1.44 from the Research Services Branch (The National Institute of Mental Health, Bethesda, MD, USA).

### 4.10. Statistical Analysis

All data were expressed as mean ± standard error of mean (S.E.M.). Statistical significance was measured by the difference among experimental groups using one-way ANOVA followed by the least significant difference post hoc multiple comparison test using GraphPad Prism 4.0 (La Jolla, CA, USA). Statistical significance was considered when *p* < 0.05.

## 5. Conclusion

The present study demonstrates that CPE’s antidiabetic, antioxidant, and renoprotective effects involve improved hyperglycemia, lipid profiles, insulin resistance, visceral fat deposition, lipid peroxidation, renal lipid accumulation, and cationic transport function. The renoprotective effects of CPE on organic cation transport might predominantly be mediated by oxidative stress/PKC activation pathways. Therefore, CPE has potential as a food supplement for preventing the progressive deterioration of renal function and delaying severe diabetic kidney disease.

## Figures and Tables

**Table 1:** Effect of CPE on selected overall nutritional and growth parameters in rats.

Parameters	ND	ND + CPE	DM	DM + CPE	DM + Met	DM + CPE + Met
Food Intake (g/day)	19.94 ± 0.67	17.99 ± 0.41	22.68 ± 1.03 *	21.21 ± 0.90	22.45 ± 0.81 *	20.66 ± 0.89
Calories Intake (kcal/day)	80.11 ± 2.68	72.17 ± 1.65	121.31 ± 5.50 *	111.90 ± 4.80 *	119.34 ± 4.40 *	119.20 ± 6.94 *
Water Intake (mL/day)	37.96 ± 2.14	44.04 ± 3.80	44.62 ± 4.08	46.92 ± 1.97	46.41 ± 1.83	40.94 ± 2.07
BW (g)	476.88 ± 9.91	448.75 ± 9.85	510.00 ± 27.12	468.33 ± 26.07	475.83 ± 32.21	464.38 ± 25.76
BW Gain (g)	136.25 ± 10.97	66.25 ± 17.13 *	130.00 ± 18.55	109.17 ± 13.44	125.83 ± 30.53	93.75 ±25.70
VFW/BW (g/100 g BW)	7.08 ± 0.35	4.74 ± 0.34 *	9.78 ± 0.88 *	7.05 ± 0.33 ^#^	8.07 ± 0.86	6.45 ± 0.66 ^#^
Relative KW	5.22 ± 0.14	5.44 ± 0.10	5.59 ± 0.37	6.13 ± 0.27	6.14 ± 0.65	5.98 ± 0.41

BW, body weight; BW gain, body weight gain; VFW/BW, visceral fat weight per body weight; relative KW, relative kidney weight (KW/BW × 100). Each value is expressed as mean ± S.E.M (*n* = 6-8). * *p* < 0.05 indicates significant differences from normal (ND), and ^#^ *p* < 0.05 indicates significant differences from diabetes (DM).

**Table 2:** Effect of CPE on selected biochemical parameters of carbohydrate and lipid metabolism as well as general kidney function in rats.

Parameters	ND	ND + CPE	DM	DM + CPE	DM + Met	DM + CPE + Met
Plasma Glucose (mg/dL)	133.25 ±3.14	132.71 ± 2.61	428.47 ± 37.27 *	237.45 ± 19.76 *^,#^	218.43 ± 28.06 *^,#^	231.10 ± 28.70 *^,#^
Plasma Triglycerides (mg/dL)	42.43 ± 4.41	42.23 ± 5.93	80.12 ± 7.93 *	43.82 ± 6.92 ^#^	50.69 ± 4.43 ^#^	40.73 ± 5.08 ^#^
Plasma insulin (ng/mL)	2.33 ± 0.35	2.81 ± 0.96	2.66 ± 0.50	1.89 ± 0.41	1.82 ± 0.35	1.97 ± 0.29
HOMA Index	20.13 ± 3.19	23.21 ± 7.97	72.19 ± 14.42 *	29.05 ± 6.94 ^#^	26.54 ± 5.35 ^#^	26.14 ± 5.46 ^#^
Estimated GFR (mL/min)	1.30 ± 0.60	1.12 ± 0.34	1.30 ± 0.27	1.31 ± 0.18	1.37 ± 0.25	1.26 ± 0.45

HOMA index, homeostasis assessment of insulin resistance index; eGFR, estimated glomerular filtration rate. Each value is expressed as mean ± S.E.M (*n* = 6–8). * *p* < 0.05 indicates significant differences from normal (ND), and ^#^ *p* < 0.05 indicates significant differences from diabetes (DM).

## Data Availability

The data presented in this study are available on request from the corresponding author.

## Sample Availability

Samples of the compounds are not available from the authors.

## Abbreviations

ABCA1	Adenosine triphosphate binding cassette (ABC) transporter A1
BW	Body weight
CGA	Chlorogenic acid
CD36	Cluster of differentiation 36
CP	*Coffea arabica* pulp
CPE	*Coffea arabica* pulp aqueous extract
cDNA	Complementary deoxyribonucleic acid
CuZn-SOD	Copper-zinc superoxide dismutase
EC	Epicatechin
ES	Estrone sulfate
GPx	Glutathione peroxidase
HOMA index	Homeostasis assessment of insulin resistance index
LXR	Liver X receptor
MDA	Malondialdehyde
MPP^+^	1-methyl-4-phenylpyridinium
ND	Normal diet
Oat1	Organic anion transporter 1
Oat3	Organic anion transporter 3
Oct2	Organic cation transporter 2
PAH	*Para*-aminohippurate
p-PKC	Phosphorylated PKC
qPCR	Quantitative real-time polymerase chain reaction
RNA	Ribonucleic acid
Na^+^-K^+^-ATPase	Sodium potassium ATPase
SREBP-1	Sterol regulatory element-binding protein 1
STZ	Streptozotocin
TBS-T	Tris-buffered saline
T2D	Type 2 diabetes
VFW	Visceral fat weight
